# Comparison of Enamel Surface Integrity after De-Bracketing as Affected by Seven Different Orthodontic Residual Cement Removal Systems

**DOI:** 10.3390/diagnostics13203284

**Published:** 2023-10-23

**Authors:** Abdullazez Almudhi, Arwa Aldeeri, Abdullah Abdulrahman A. Aloraini, Ahmed Ibrahim M. Alomar, Meshari Saad M. Alqudairi, Osama Abdullah A. Alzahrani, Elzahraa Eldwakhly, Sarah AlMugairin

**Affiliations:** 1 Department of Pediatric Dentistry and Orthodontics, College of Dentistry, King Saud University, Riyadh 11545, Saudi Arabia; abalmudhi@ksu.edu.sa; 2 College of Dentistry, King Saud University, Riyadh 11545, Saudi Arabia; 3 Department of Clinical Dental Sciences, College of Dentistry, Princess Nourah Bint Abdulrahman University, Riyadh 11671, Saudi Arabia; 4 Department of Preventive Dental Sciences, College of Dentistry, Princess Nourah Bint Abdulrahman University, Riyadh 11671, Saudi Arabia

**Keywords:** orthodontic adhesive, orthodontic brackets, dental enamel, SEM, dental debonding

## Abstract

This study used seven different adhesive removal systems to evaluate and compare enamel surface integrity, heat generation, and time consumed during residual cement removal after de-bracketing. The sample size was 140 human premolars. Teeth were cleaned, mounted, and prepared for orthodontic bracket bonding. Brackets were then debonded using bracket-removing pliers. Teeth were randomly assigned to seven groups based on the residual cement removal system: Group 1: Stainbuster bur, Group 2: Renew diamond bur #129, Group 3: Renew carbide bur, Group 4: OneGloss Complete system, Group 5: Sof-Lex system, Group 6: Enhance Finishing and PoGo Polishing complete kit, and Group 7: Renew friction grip points. The enamel surface was evaluated for roughness before bracketing and after residual cement removal using surface profilometry. The time taken for cement removal was recorded using a digital timer, and heat generation was measured using a laser thermometer before and after cement removal. One-way ANOVA compared the pre- and post-values for enamel surface roughness, temperature, and time consumed. When comparing the difference between the post- and pre-finishing roughness using one-way ANOVA, the Renew diamond bur produced the roughest enamel surface post-removal with a mean of 4.716 μm, while the Sof-Lex recorded the lowest at 0.760 μm. The highest mean temperature was recorded with the Stainbuster bur at 5.545 °C, and the lowest temperature was recorded with the Enhance bur at 2.260 °C. The time for cement removal was the shortest with the Enhance bur at 12.2 s, whereas the time was the longest with the Renew diamond bur at 30.4 s. In conclusion, all the residual cement removal systems used in this clinically simulated study were not able to restore the original enamel surface smoothness. However, the 3M Sof-Lex produced the lowest enamel roughness but with more time consumption and heat generation. When selecting the best residual cement removal system to be used, clinicians should weigh the merits and demerits of each system based on the clinical judgement of the operator.

## 1. Introduction

Orthodontists face a strategic challenge following the completion of orthodontic cases treated with fixed appliances. The challenge orthodontists face revolves around the restoration of the enamel surface to its original texture, appearance, smoothness, and condition to match its pre-orthodontic therapy state. A roughened enamel surface not only inhibits proper cleaning but also promotes plaque retention, bacterial accumulation, and the formation of stains, which negatively impacts the aesthetic appearance of the teeth [1,2]. Therefore, the primary objective in orthodontic treatment is to preserve the enamel surface as much as possible while avoiding inadvertent damage and minimizing aesthetic changes as much as permissible. Orthodontic brackets being bonded and debonded to the teeth involve multiple steps that, if not executed correctly, can result in enamel damage and alteration in its original morphology resulting in about 10–20% wear of the enamel surface [3,4]. De-bracketing and removal of residual orthodontic adhesive is often a complex task that may involve the mechanical removal of enamel, posing a risk to healthy dental structure and causing irreversible enamel damage [5]. Restoring the enamel back to its original structure, as it existed prior to orthodontic treatment with fixed appliances, can be accomplished via the complete removal of post de-bracketing adhesive [6]. However, this removal process is rather challenging, as it often affects the enamel surface, resulting in increased roughness, enamel cracking, and discoloration due to lingering adhesive residues and alterations in optical properties, such as how light reflects off the enamel crystals [7]. Moreover, it can lead to anatomical changes that promote bacterial retention and expose enamel prisms to the oral environment, causing decreased enamel resistance to plaque organic acids, facilitating enamel demineralization [8,9]. Consequently, the removal of orthodontic brackets and any residual adhesive materials becomes a critical post-orthodontic procedure. Bracket removal should aim not only to detach the bracket base from the tooth but also to eliminate any remaining adhesive in order to bring the enamel back to its pre-treatment state [10]. While some level of scarring on the enamel surface during adhesive removal is often inevitable, the extent of damage can be considerably minimized through the selection of appropriate adhesive removal techniques [11]. In this context, various methods are employed to remove cement after orthodontic treatment, each with varying degrees of effectiveness, but often leading to enamel roughness. These methods encompass the use of scalers (both manual and ultrasonic), intraoral sandblasting, burs (such as diamond, tungsten, stainless steel, and composite burs), and lasers [12,13]. Different factors, including the bur type, its rotational speed, the number of blades, and the material composition, influence the extent of the enamel damage during adhesive removal [5,10,14]. The gold-standard bur for removing residual cement is a tungsten carbide bur with either 12 or 20 flutes [15,16]. According to a recent systematic review, tungsten carbide burs proved to be exceptionally efficient and time-saving for residual cement removal when compared to Sof-Lex discs, ultrasonic tools, hand instruments, rubber instruments, or composite burs. Furthermore, the tungsten carbide burs were found to be less abrasive and less destructive than Arkansas stones, green stones, diamond burs, steel burs, and lasers [17].

Recently, four conservative finishing and polishing systems have gained popularity among orthodontists: the Sof-Lex (3M ESPE, St. Paul, MN, USA), the Enhance Finishing and PoGo Polishing system (Dentsply Sirona, Charlotte, NC, USA), the OneGloss Complete system (Shofu Dental, Kyoto, Japan), and Stainbuster (Abrasive Technology, Lewis Center, OH, USA) [18,19,20,21,22].

Several techniques can be employed to assess enamel roughness, including scanning electron microscopy (SEM), stereomicroscopy, contact profilometry, non-contact white-light three-dimensional (3D) profilometry, and atomic force microscopy [12,23]. Research efforts continue to explore improved adhesive removal methods that effectively eliminate residual resin while preserving the enamel surface as closely as possible to its original thickness [24,25]. Currently, no agreed-upon accepted clinical protocol can entirely remove residual adhesive cement remnants without the potential risk of damaging the enamel surface following orthodontic bracket debonding [5,17].

According to a review of the recent scientific literature, no study has comprehensively compared the following seven contemporary finishing systems used for the removal of residual orthodontic adhesive to assess their efficiency on a single platform; the Sof-Lex [3M ESPE], the Enhance Finishing and PoGo Polishing system [Dentsply Sirona], the OneGloss Complete system [Shofu Dental], Stainbuster [Abrasive Technology], the Renew friction grip points [Reliance Orthodontics, Itasca, IL, USA], the Renew Finishing System with a carbide bur [Reliance Orthodontics], and the Renew Finishing System with a diamond bur #129 [Reliance Orthodontics]. Consequently, this study aimed to evaluate and compare the integrity of the enamel surface, heat generation, and time consumed during the process of residual cement removal after de-bracketing using seven different contemporary adhesive removal systems to find the most effective and efficient method of removing residual cement. This study’s tested null hypothesis postulates that there are no significant variations between the seven different examined adhesive removal systems regarding the quality of enamel surface roughness, the heat generated within the teeth, or the procedure duration timing.

## 2. Materials and Methods

This study was conducted in accordance with the Declaration of Helsinki and approved by the Research Ethics Committee of the Research Center, King Saud University (E-21-6414).

Sample collection: Freshly extracted for orthodontic treatment, sound human premolar teeth were collected.

The following inclusion criteria were used during sample collection: caries-free, hypoplastic-defect-free, and restoration-free premolars were included. Fractured or cracked teeth and teeth with morphological abnormalities were excluded. The collected teeth were stored in saline at 7 °C up until the time of testing.

A sample of 140 human premolars (*n* = 140) was used according to the sample size determination: At alpha = 0.05 with effect (1-b) = 0.9 (power) and size ES = 0.4, the total sample size should be at least 140 and 20 selected randomly for each group.

The teeth were randomly divided into seven groups (*n* = 20 teeth per group), and each group was assigned to a different finishing system, Table 1.

### 2.1. Surface Roughness Initial Measurement (Pre-Bracket Bonding)

**Figure 1:**
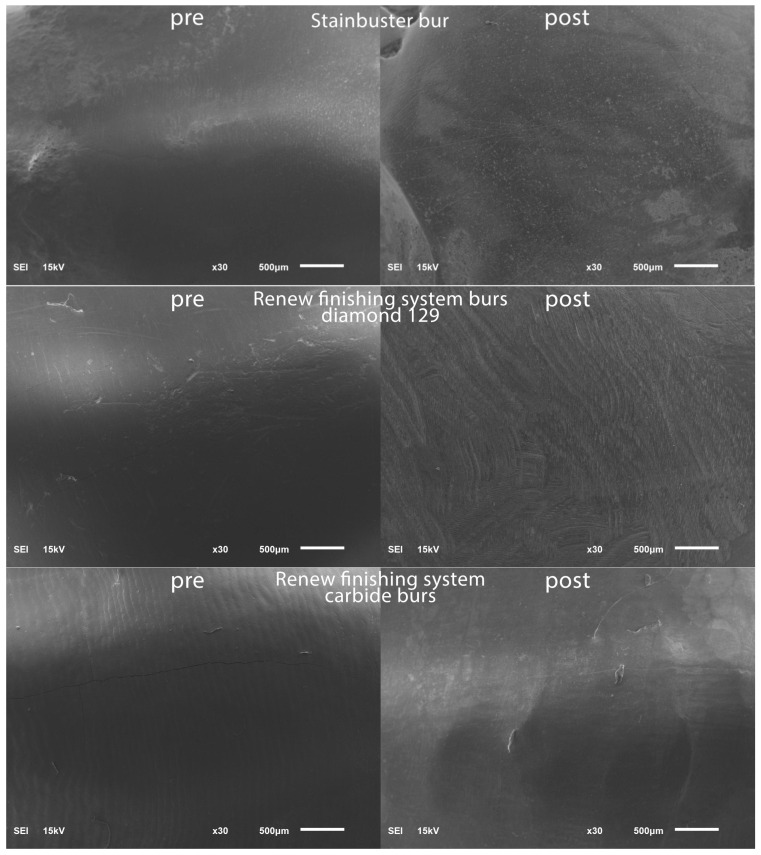

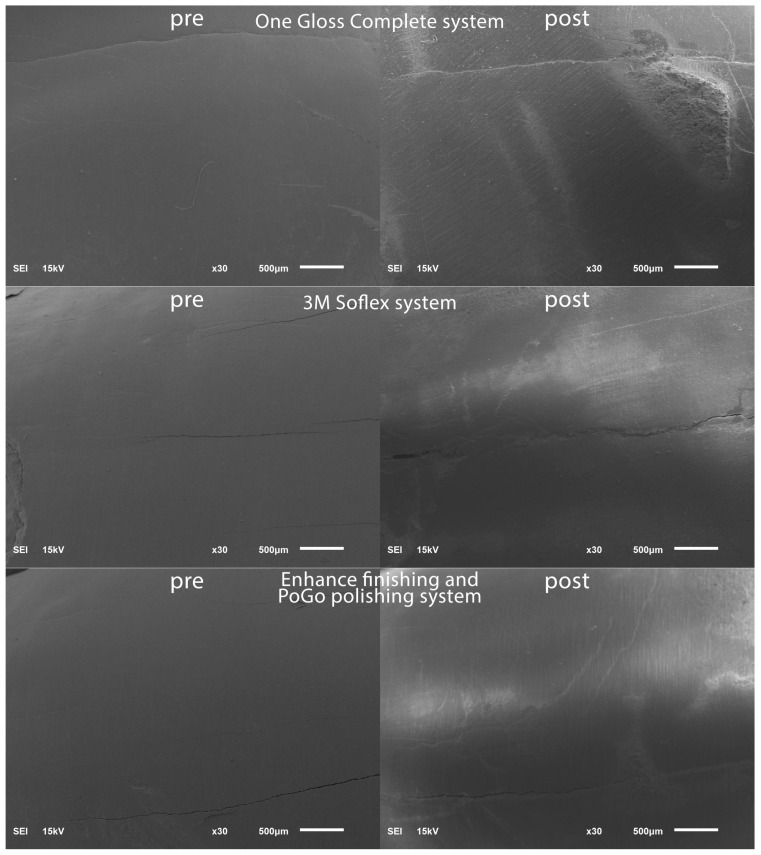

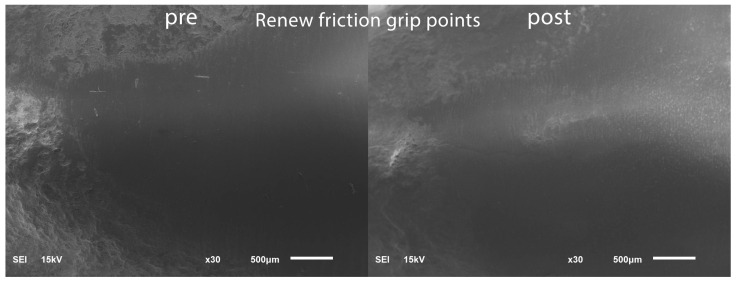
SEM images pre-bracket bonding and post-bracket debonding.

To establish a baseline for the surface roughness data, the teeth were positioned within a vinyl polysiloxane putty mold in preparation for scanning using a scanning electron microscope (SEM) (JSM 6610LV; Jeol, Tokyo, Japan). The SEM parameters employed included 15 kV voltage and a 10 mm working distance. To highlight specific regions of interest on the facial tooth surface, magnifications of 30× were chosen and representative SEM scans were captured in the pre-bracket bonding state; see Figure 1.

Before surface profilometry, the teeth were prepared by submerging them in an ultrasonic cleaner containing distilled water for 15 min to remove any debris. They were then air-dried for 60 s. Subsequently, the samples were placed in the sample stage of a Q150R coating machine (QuorumTech, Laughton, UK) to apply a thin 10 nm gold film onto the sample surface during a 1 min run. A Contour GT-K 3D optical microscope (Bruker, Billerica, MA, USA), utilizing 3D non-contact surface metrology with interferometry, was employed for surface profilometer characterization and measurement.

The samples were subjected to vertical scan interferometry using a 5× Michelson magnification lens with a 1 mm × 1 mm field of view, Gaussian regression filter, scan speed of 1×, and thresholding set at 4. The samples were positioned on the stage and manually adjusted to obtain an image on the monitor screen. The microscope was controlled using Vision 64 software (Bruker), which managed the instrument settings, data analysis, and graphical output. The measurement was conducted via vertical scanning interferometry, making use of a broadband (typically white) light source suitable for measuring objects with rough surfaces and adjacent pixel height differences exceeding 135 nm. Each sample underwent three scans at different intervals, and the results were averaged to determine roughness.

### 2.2. Preparation of Teeth for Bracket Bonding

For tooth preparation, a 37% phosphoric acid gel was applied to the buccal enamel surface for 15 s, followed by a constant water rinse for 10 s and a 1 -s drying period using an oil-free air jet. Subsequently, to ensure uniform adhesive distribution, the adhesive (Transbond XT. adhesive, 3M Unitek, Monrovia, CA, USA) was applied to the mid region of the bracket’s base (3M Victory Series Low Profile Bracket System), and proper pressure was applied to extrude any excess cement. The excess adhesive was removed from around the bracket using a dental explorer. The Transbond XT. adhesive was light cured on all sides of the bracket using an LED light cure unit for 20 s, as per the manufacturer recommendation. One operator carried out the bonding procedure of all brackets to the dental surfaces for the seven tested premolar groups.

The brackets were ultimately removed using a bracket removal plier (3M Unitek removing pliers). Then, each group was administered for residual cement removal using one of the seven tested residual cement removal systems; see Table 1.

A single individual (an orthodontics specialist) performed the remnant cement removal procedure using each of the seven tested residual cement removal systems to ensure standardization and reduce variability. This procedure was performed over a period of 3 days, and the manufacturer’s instructions were strictly adhered to for each cement removal system.

The duration of each residual cement removal technique was recorded using a digital timer, and heat generation was measured using a laser thermometer before and after cement removal. The collected data were statistically analyzed.

### 2.3. Surface Roughness Final Measurement (Post-Bracket Debonding)

Post-residual cement removal, enamel roughness measurements were performed in a similar routine to the pre-operative enamel surface roughness measurements and representative SEM scans were captured post-bracket debonding; see Figure 1.

### 2.4. Statistical Analysis

The data were statistically analyzed using the IBM-SPSS software (Version 26.0, 2019). One-way ANOVA compared the pre- and post-residual adhesive removal enamel surface roughness values, pre- and post-residual adhesive removal teeth temperature values, and the time consumed for residual adhesive removal.

## 3. Results

No significant difference was found in the baseline roughness between the seven groups (*p* = 0.201); see Table 2.

**Figure 2:**
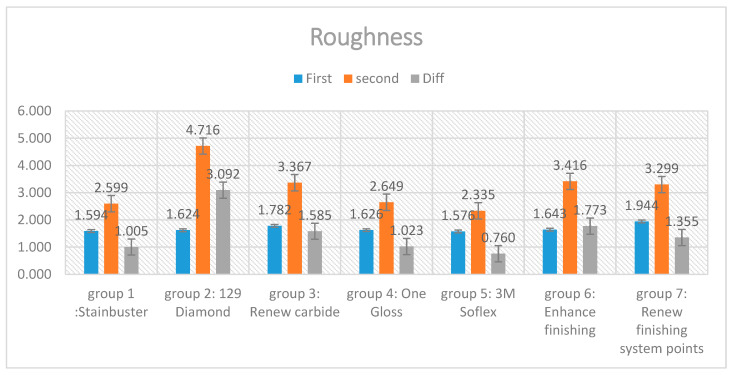
Roughness values.

When comparing the difference between the post- and pre-finishing roughness using one-way ANOVA, the mean value of the Renew diamond bur (4.716 μm) was the highest, followed by the Enhance bur (1.773 μm), the Renew carbide bur (1.585 μm), the Renew finishing points (1.349 μm), the OneGloss system (1.023 μm), Stainbuster (1.005 μm), and the Sof-Lex (0.760 μm), Figure 2, Table 3.

There was a significant difference between the pre- and post-finishing temperature using one-way ANOVA (*p* < 0.000); see Table 4. 

**Figure 3:**
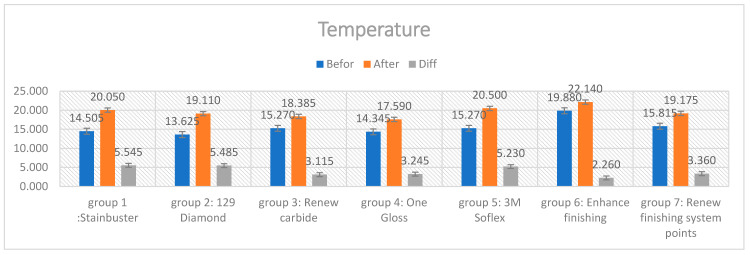
Temperature values.

The mean temperature difference with the Stainbuster (5.545 °C) was the highest, followed by the Renew diamond bur (5.485 °C), the Sof-Lex (5.230 °C), the Renew Finishing Points (3.360 °C), the OneGloss (3.245 °C), the Renew carbide bur (3.115 °C), and the Enhance bur (2.260 °C), Figure 3, Table 5.

**Figure 4:**
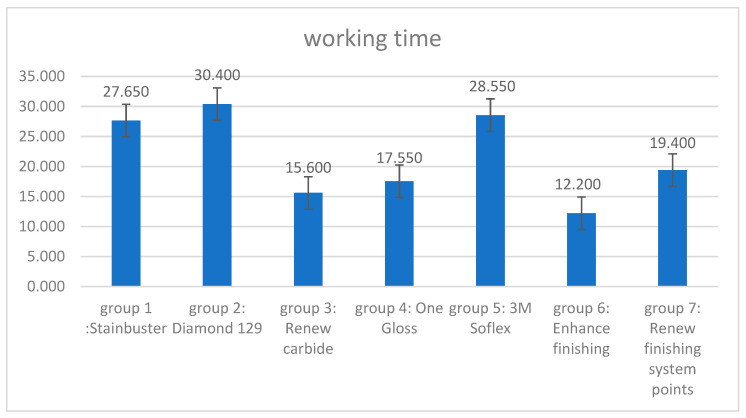
Working time values.

The Enhance bur took the least amount of working time to remove residual cement (12.2 s), followed by the Renew carbide bur (15.6 s), the OneGloss (17.5 s), the Renew Finishing System Points (19.4 s), the Stainbuster (27.6 s), the Sof-Lex (28.5 s), and the Renew diamond bur (30.4 s), Figure 4**,** Table 6.

## 4. Discussion

This study investigated the impact of seven different evidence-based approaches to orthodontic residual cement removal systems on enamel surface after de-bracketing regarding the three variables of enamel surface roughness, temperature of teeth in terms of the heat generated during residual cement removal, and the working time taken for the removal process. The results of this study recorded significant differences between the seven tested residual cement removal systems in terms of the three tested variables: induced enamel surface roughness; heat generation within the tooth during the residual cement removal process; as well as the time taken for completion of the procedure. Accordingly, the tested null hypothesis was rejected.

The responsibility of orthodontists in the selection of an appropriate technique for adhesive removal after bracket debonding with the aim of minimizing iatrogenic damage and scratches on the enamel surface, as well as preservation of enamel surface smoothness, is highlighted in this research [26]. Orthodontists are advised to choose a protocol for adhesive remnant removal based on scientific evidence in order to accomplish a conservative and effective outcome post-fixed orthodontic treatment.

Regarding the results of the first tested variable, enamel surface roughness, no significant differences were recorded between the teeth assigned to the seven different tested groups in the original enamel condition before bracket bonding. Similar results were reported by other studies [27,28]. Various studies have reported different degrees of enamel loss during the removal of residual adhesive material, ranging from a few micrometers to around 50 μm [28,29,30,31]. Enamel thickness is stated to range from 1500 μm to 2000 μm; accordingly, it was reported that enamel loss of around 60 μm is not considered detrimental to the tooth [32]. Nonetheless, preserving the fluoride enriched surface layer of enamel is crucial when removing residual composite, due to the fact that the highest concentration of fluoride lies at the surface and decreases immensely after the first 20 μm of enamel thickness [32,33]. All of the seven tested residual cement removal systems in this study recorded post-de-bracketing surface roughness values well below the 20 μm range. 

This research indicated that the different methods used for the removal of residual adhesive resulted in different degrees of enamel loss; this was in accordance with other studies [34,35]. It was observed in this study that all seven tested finishing systems had an effect on the enamel tooth surface, and none of the residual cement removal systems succeeded in maintaining the original shape and smoothness of the enamel surface compared to the enamel condition prior to the de-bracketing condition. The results of this study showed that the post-de-bracketing enamel surface roughness was constantly higher than the pre-de-bracketing roughness in all groups, indicating that no residual cement removal method was fully capable of restoring the enamel surface back to its original state. This was in accordance with several studies that proved that none of their tested remnant cement removal systems restored the enamel surface roughness to the original pretesting status [8,9,18,24,28,31]. This could be attributed to the fact that adhesive resin tags are capable of penetrating the enamel up to 50 μm during enamel conditioning and bonding procedures [31].

Significant differences were noticed between the seven tested residual cement removal systems in terms of the surface roughness introduced into the enamel surface. The Sof-Lex system group demonstrated the smoothest post-finishing enamel surface. The Sof-Lex was found to significantly reduce surface roughness, as evidenced by the lowest enamel roughness measurements (2.335 μm), compared to the other tested methods. The Stainbuster system recorded the second lowest enamel roughness measurements (2.599 μm). The OneGloss system recorded the third lowest enamel roughness measurements (2.649 μm). Renew Finishing System Points recorded the fourth lowest enamel roughness measurements (3.299 μm). The Renew carbide bur system recorded the fifth lowest enamel roughness measurements (3.367 μm). The Enhance Finishing and PoGo Polishing system recorded the sixth lowest enamel roughness measurements (3.416 μm), whereas the Renew #129 diamond bur system was found to produce the highest levels of roughness on the enamel surface (4.716 μm), as evidenced by the surface profilometry and scanning electron microscopy (SEM) results. In related context, Bansal et al. [28] suggested that the Sof-Lex system ranked second after the Mylar matrix in terms of smoothing, while Shah et al. [24] reported that the Sof-Lex system also secured the second position, following the Enhance Finishing system.

Concerning the results of the second tested variable, the increase in tooth temperature following adhesive cement remnant removal, it was found that there was a noticeable increase in tooth temperature following the finishing procedure. Similar to the enamel surface roughness results obtained in this study, significant differences were noticed between the seven tested residual cement removal systems in terms of the heat generated during the removal of residual cement and finishing procedures. This is in agreement with other studies that reported increase in tooth temperature due to heat generation during the residual cement removal procedure [31,32,36]. The Stainbuster bur system group exhibited the highest degree of heat generation with a difference between the tooth temperature before and after residual cement removal of 5.545 °C. The Renew #129 diamond bur system recorded the second highest degree of heat generation (5.485 °C). The Sof-Lex system group demonstrated the third highest degree of heat generation (5.230 °C). The Renew Finishing system points group displayed the fourth highest degree of heat generation (3.360 °C). The OneGloss system group presented the fifth highest degree of heat generation (3.245 °C), while both the Renew carbide bur and the Enhance Finishing and PoGo Polishing systems recorded the lowest degree of heat generation at 3.115 °C and 2.260 °C, conversely.

Relating to the results of the third tested variable, the working time and efficiency of the seven tested residual cement removal systems, it was reported that the recorded working time of the procedure of the removal of the remaining cement varied across the seven tested systems. This study’s results of the different timings recorded for the different residual cement removal systems were in agreement with other studies that reported variations in the residual adhesive removal procedure according to the system used [37,38]. The duration of the procedure of removal of the remaining cement was the longest with the Renew #129 diamond bur system requiring 30.40 s for residual cement removal. The Sof-Lex system group reported the second longest operating time (28.55 s). The Stainbuster bur system group recorded the third longest operating time (27.65 s). The Renew Finishing system points group presented the fourth longest operating time (19.40 s). The OneGloss system group showed the fifth longest operating time (17.55 s). The Renew carbide bur group revealed the sixth longest operating time (15.60 s). The Enhance Finishing and PoGo Polishing systems proved to be the least time-consuming, taking only 12.2 s for the whole duration of residual cement removal procedure.

The results of this study align with the current literature, documenting that none of the available residual cement removal techniques can completely remove cement remnants from dental tooth surfaces without causing some enamel damage.

The limitations of this study arise from the in vitro study design that prevents the evaluation of the dentinal reaction and pulpal response to the heat generated during the residual orthodontic adhesive removal.

## 5. Conclusions

Within the limitations of this study, all seven orthodontic residual cement removal methods were clinically satisfactory for residual orthodontic adhesive removal.

Nevertheless, none of the evaluated systems were able to completely restore the original enamel surface smoothness.

Yet, all seven orthodontic residual cement removal methods yielded a clinically suitable enamel surface roughness, the greatest range of which was well below the 20 μm range. 

The 3M Sof-Lex system exhibited the lowest enamel roughness, but at the expense of being more time-consuming and generating higher heat levels compared to the six other residual cement removal systems.

## Figures and Tables

**Table 1:** Group Distributions.

Group	*n*
Group 1: Stainbuster (Abrasive Technology)	20
Group 2: Renew diamond bur #129 (Reliance Orthodontics)	20
Group 3: Renew carbide burs (Reliance Orthodontics)	20
Group 4: OneGloss Complete system (Shofu Dental)	20
Group 5: Sof-Lex system (3M ESPE),	20
Group 6: Enhance Finishing and PoGo Polishing complete kit (Dentsply Sirona)	20
Group 7: Renew friction grip points (Reliance Orthodontics)	20

**Table 2:** Baseline Roughness Values.

Baseline Roughness	Sum of Squares	df	Mean Square	F	*p*
Between Groups	2.112	6	0.352	1.447	0.201
Within Groups	32.358	133	0.243		

**Table 3:** Using ANOVA to compare groups by the average roughness.

Roughness					Difference		Paired *t*-Test	*p*-Value Diff of Group	MCT (Tukey HSD)						
	First		Second						Group 1: Stainbuster	Group 2: Diamond	Group 3: Renew Carbide	Group 4: One Gloss	Group 5: 3M Soflex	Group 6: Enhance Finishing	Group 7: Renew Finishing System Points
Group	Mean	Std. Deviation	Mean	Std. Deviation	Diff	SD Dif	*p*-Value								
Group 1: Stainbuster	1.594.	0.586	2.599	1.109	1.005	0.850	0.000	0.000	1						
Group 2: Renew #129 diamond	1.624	0.443	4.716	1.074	3.092	1.156	0.000		0.000	1					
Group 3: Renew carbide	1.782	0.512	3.367	1.015	1.585	1.116	0.000		0.474	0.000	1				
Group 4: OneGloss	1.626	0.530	2.649	0.706	1.023	0.730	0.000		1.000	0.000	0.513	1			
Group 5: 3M Sof-Lex	1.576	0.428	2.335	0.603	0.760	0.588	0.000		0.984	0.000	0.100	0.976	1		
Group 6: Enhance Finishing	1.643	0.418	3.416	0.995	1.773	0.883	0.000		0.155	0.001	0.996	0.176	0.018	1	
Group 7: Renew Finishing System Points	1.944	0.512	3.299	1.195	1.355	1.217	0.000		0.921	0.000	0.987	0.938	0.470	0.810	1

**Table 4:** Pre- and post-residual cement removal teeth temperature means.

Temperature Difference	Sum of Squares	df	Mean Square	F
Between Groups	217.747	6	36.291	6.316
Within Groups	764.268	133	5.746	

**Table 5:** Using ANOVA to compare groups by the average temperature.

Temperature					Difference		Paired *t*-Test	*p*-Value Diff of Group	MCT (Tukey HSD)						
	Before		After												
									Group 1: Stainbuster	Group 2: Diamond	Group 3: Renew carbide	Group 4: OneGloss	Group 5: 3M Sof-Lex	Group 6: Enhance Finishing	Group 7: Renew Finishing System Points
Group	Mean	Std. Deviation	Mean	Std. Deviation	Diff	SD Dif	*p*-Value								
Group 1: Stainbuster	14.505	1.209	20.050	1.963	5.545	1.862	0.000	0.000	1						
Group 2: Renew #129 diamond	13.625	0.596	19.110	1.349	5.485	1.626	0.000		1.000	1					
Group 3: Renew carbide	15.270	0.716	18.385	1.657	3.115	1.535	0.000		0.027	0.035	1				
Group 4: OneGloss	14.345	1.599	17.590	2.815	3.245	2.160	0.000		0.045	0.056	1.000	1			
Group 5: 3M Sof-Lex	15.270	0.954	20.500	1.620	5.230	2.094	0.000		1.000	1.000	0.085	0.129	1		
Group 6: Enhance Finishing	19.880	3.837	22.140	2.000	2.260	3.964	0.000		0.001	0.001	0.918	0.851	0.003	1	
Group 7: Renew Finishing System Points	15.815	1.340	19.175	2.750	3.360	2.645	0.000		0.067	0.082	1.000	1.000	0.180	0.770	1

**Table 6:** Using ANOVA to compare groups by the average working time.

Working Time					95% Confidence Interval for Mean		MCT						
							Group 1: Stainbuster	Group 2: Diamond	Group 3: Renew Carbide	Group 4: One Gloss	Group 5: 3M Soflex	Group 6: Enhance Finishing	Group 7: Renew Finishing System Points
Group	*n*	Mean	Std. Deviation	*p*-Value	Lower Bound	Upper Bound							
Group 1: Stainbuster	20	27.650	15.295	0.000	5.545	1.862	1						
Group 2: Renew #129 Diamond	20	30.400	6.793		5.485	1.626	0.934	1					
Group 3: Renew carbide	20	15.600	3.409		3.115	1.535	0.000	0.000	1				
Group 4: OneGloss	20	17.550	6.177		3.245	2.160	0.002	0.000	0.988	1			
Group 5: 3M Sof-Lex	20	28.550	5.799		5.230	2.094	1.000	0.991	0.000	0.001	1		
Group 6: Enhance finishing	20	12.200	4.060		2.260	3.964	0.000	0.000	0.835	0.360	0.000	1	
Group 7: Renew Finishing System Points	20	19.400	8.696		3.360	2.645	0.025	0.001	0.751	0.991	0.008	0.079	1

## Data Availability

The data presented in this study are available on request from the corresponding author.
